# Early Prognostic Factors in Multiple Sclerosis: Clinical and Therapeutic Implications

**DOI:** 10.3390/medicina62030475

**Published:** 2026-03-02

**Authors:** Katarzyna Maciejowska-Szydło, Przemysław Puz

**Affiliations:** Department of Neurology, Faculty of Health Sciences in Katowice, Medical University of Silesia, 40-635 Katowice, Poland; kmaciejowska@gcm.pl

**Keywords:** multiple sclerosis, prognostic factors, magnetic resonance imaging, biomarkers, neurofilaments, disease-modifying therapy, disability progression

## Abstract

*Introduction*: Multiple sclerosis (MS) is a chronic, inflammatory, demyelinating disease of the central nervous system with a highly heterogeneous clinical course. Early identification of patients at risk of aggressive disease progression is crucial for optimizing therapeutic strategies, including eligibility for highly effective treatments. *Objective*: The aim of this review was to synthesize current data on prognostic factors in multiple sclerosis, with particular emphasis on their significance in the early stages of the disease and potential clinical implications. *Methods*: A narrative systematic review of the literature was conducted, including observational studies, cohort studies, meta-analyses, and systematic reviews on the natural course of MS, prognostic factors, and clinical, neuroimaging, and laboratory biomarkers. We comprehensively reviewed PubMed and Scopus databases, focusing on English-language publications. Study selection prioritized longitudinal studies and meta-analyses with clear outcome definitions and sufficient follow-up. Formal quality scoring was not applied due to the narrative design of the review. *Results*: Key adverse prognostic factors include older age at onset, polysymptomatic onset, high relapse activity in the first years, incomplete remission after relapses, and the primary progressive form. Magnetic resonance imaging features, including the number and location of T2 lesions, contrast activity, the presence of spinal cord lesions, PRLs and SELs, and severe brain atrophy, also have significant predictive value. Increasing importance is being attached to laboratory biomarkers, such as oligoclonal bands, light neurofilaments, free kappa light chains, and GFAP. *Conclusions*: An integrated assessment of clinical, neuroimaging, and laboratory factors enables more effective risk stratification in patients with newly diagnosed MS. Early identification of an unfavorable prognostic profile may provide a basis for individualizing treatment and considering the use of highly effective therapies early in the course of the disease.

## 1. Introduction

Multiple sclerosis (MS) is a chronic, inflammatory, demyelinating immune disease of the central nervous system that leads to progressive neurological disability, especially in young people and those of working age. Despite significant advances in diagnosis and the development of disease-modifying therapies, MS remains a highly heterogeneous disease, and predicting individual prognosis at an early stage of the disease remains a significant clinical challenge [1,2]. Although prognostic factors in multiple sclerosis have been thoroughly investigated, it is necessary to integrate heterogeneous clinical, imaging and laboratory data into a coherent framework that can be directly applied to early therapeutic decision-making. The existing literature often focuses on individual markers in isolation, while their combined translational value for everyday clinical practice is less clearly defined.

A characteristic feature of MS is the coexistence of inflammatory, demyelinating, and neurodegenerative processes, the relative severity of which varies with the duration of the disease and the age of the patient. Some patients experience a mild, slowly progressive course, while others experience rapid accumulation of disability, often within the first few years after diagnosis. This variability in course means that therapeutic decisions made early in the disease are critical for long-term clinical outcomes [1,2,3].

In recent years, increasing attention has been paid to the importance of early therapeutic intervention, including the use of highly effective therapies at the onset of treatment. This strategy may be more effective in reducing inflammatory activity and potentially influencing the long-term course of the disease, but its optimal use requires precise identification of patients with an unfavorable prognostic profile. In clinical practice, there is still a lack of clear algorithms for reliable risk stratification in patients with newly diagnosed MS [4,5].

Available data indicate that the prognosis in MS is determined by a complex interaction of demographic, clinical, neuroimaging, and laboratory factors. Early relapse activity, the nature of initial symptoms, the dynamics of disability progression, magnetic resonance imaging features, and the presence of specific biomarkers may provide important prognostic information. However, most available evidence derives from observational and registry-based studies, which are inherently prone to selection bias and heterogeneity in outcome definitions. The results of individual studies are often heterogeneous, and the significance of many potential markers remains a subject of debate [2,4,6,7].

The aim of this review is to present the current data on prognostic factors in multiple sclerosis, with particular emphasis on their significance in the early stages of the disease. This review aims to provide a synthetic and critical assessment of the available data and to identify factors that may have practical application in everyday clinical practice, supporting the individualization of treatment and decision-making regarding the early use of highly effective therapies.

### The Natural Course of Multiple Sclerosis

The natural course of multiple sclerosis is characterized by significant clinical variability, both in terms of inflammatory activity and the rate of disability progression. Long-term cohort studies and registry-based analyses confirm that early disease activity strongly influences long-term outcomes, even in the treatment era. Some patients experience a mild course of the disease, characterized by a small number of relapses and slow progression, while others experience a rapid accumulation of neurological deficits in the first years after the onset of symptoms. It is estimated that the mild form of MS occurs in approximately 15–40% of patients, depending on the criteria used and the duration of observation [8]. While traditionally a subgroup of patients has been classified as having a mild or benign disease course, contemporary data suggest that this designation is unstable over time and may underestimate future disability accumulation. This has important implications for early prognostic assessment and treatment planning. Importantly, modern natural history studies increasingly emphasize age-related mechanisms of progression, supporting the concept that inflammatory and neurodegenerative processes coexist from the earliest disease stages [9].

It is most often defined as the persistence of a low degree of disability (EDSS < 3.0) after 10–15 years of disease duration. However, long-term data indicate that even in patients initially classified as “mild,” a significant percentage experience progression over time, including the development of secondary progressive forms and significant mobility limitations. This undermines the usefulness of the concept of mild MS as a stable prognostic category [10].

In contrast to benign phenotypes, highly active forms of multiple sclerosis are characterized by rapid disability progression, high relapse activity, and significant radiological activity. Clinical criteria suggesting an aggressive course include, among others, reaching an EDSS score of ≥4.0 within the first few years of the disease, frequent relapses without full remission, and lack of response to standard disease-modifying therapy [11]. The diagnosis of such phenotypes is of significant prognostic and therapeutic importance.

The results of long-term cohort studies and analyses of the natural history of MS suggest that the rate of disability progression is largely determined by the patient’s age, regardless of the clinical phenotype at the onset of the disease. Both patients with an initial relapsing-remitting course and those with a primary progressive form reach subsequent disability milestones (EDSS 3.0; 6.0; 7.0) at a similar age, which supports the concept of MS as a disease with common mechanisms underlying progression [12].

Epidemiological data indicate that in untreated patients, the average time to reach significant disability (EDSS 6.0) is several years, while the total survival time from the onset of the first symptoms averages 30–40 years [10,12]. Mortality rate in MS is significantly higher than in the general population, especially in patients with advanced disability, and the causes of death are most often indirect and include infectious complications and comorbidities [13,14].

Understanding the natural course of multiple sclerosis is the foundation for identifying prognostic factors and rationally planning therapeutic strategies. In the context of modern treatment options, early assessment of risk factors of aggressive disease progression is particularly important, as it allows for individualized treatment and potential modification of the natural course of MS.

## 2. Literature Search and Review Methodology

This article was designed as a narrative review aimed at synthesizing clinically relevant evidence on early prognostic factors in multiple sclerosis. The literature search was performed using PubMed and Scopus databases, covering publications up to 2025. Keywords included combinations of “multiple sclerosis,” “prognosis,” “risk factors,” “MRI,” “biomarkers,” and “disability progression.”.

Inclusion criteria comprised original observational studies, cohort studies, meta-analyses, and systematic reviews focusing on prognostic factors identifiable at diagnosis or in the early stages of MS. Case reports and studies lacking clear clinical or radiological outcomes were excluded. Given the heterogeneity of study designs and endpoints, no formal quality scoring system was applied; instead, emphasis was placed on study size, follow-up duration, and consistency of findings across independent cohorts.

## 3. Prognostic Factors in Multiple Sclerosis

Achieving therapeutic goals in multiple sclerosis, such as no evidence of disease activity (NEDA), requires early and accurate selection of disease-modifying treatment tailored to the individual patient profile. In clinical practice, prognostic factors available at the time of diagnosis or in the first years of the disease, are gaining importance because they allow to predict the risk of disability progression and aggressive MS course.

Prognostic factors can be divided into four main groups: demographic, clinical, neuroimaging and laboratory. While none of these factors alone provide sufficient predictive accuracy, their combined assessment offers a more robust approach to early risk stratification. Importantly, the strength of evidence supporting individual prognostic factors varies considerably, and direct comparisons between studies are often limited by methodological heterogeneity. This multidimensional perspective represents a key conceptual shift from traditional single-marker prognostication toward integrated, patient-centered risk assessment.

### 3.1. Demographic Factors

Gender, age at onset, ethnicity, and environmental factors are among the earliest available parameters assessed in patients with multiple sclerosis. The disease is more common in women, indicating the important role of hormonal and immunological factors in the pathogenesis of MS. However, the impact of gender on the further course of the disease remains unclear. Some studies suggest that being male is associated with a faster progression of disability, while other analyses do not confirm significant prognostic differences between the sexes [8,15,16,17,18]. Data from cohorts of patients with clinically isolated syndrome indicate a comparable risk of conversion to clinically definite MS and disability progression in women and men [19]. There are data suggesting that female sex is associated with higher relapse activity (17.7% higher) although combined with younger age at onset of symptoms can be associated with EDSS improvement after treatment [20,21].

The importance of age at onset is more consistently confirmed. Older age at onset is associated with a shorter time to reach significant disability milestones, such as EDSS 4.0 or 6.0 [22]. At the same time, patients who develop the disease at a younger age may achieve a higher degree of disability in the long term, highlighting the complex influence of age on the course of MS [23,24].

Population studies have also shown differences in disease progression depending on ethnicity. African American, Hispanic and Latino patients have been observed to accumulate disability more rapidly than the Caucasian population, even after taking into account socioeconomic factors and access to healthcare [25,26,27,28].

Comorbidities and lifestyle factors also have a significant impact on prognosis. The presence of cardiovascular diseases and their risk factors, such as hypertension, diabetes, and dyslipidemia, is associated with faster progression of disability [29]. Excessive body weight at a young age and smoking are additional factors that adversely affect the course of the disease, with smoking being associated with both greater inflammatory activity and accelerated EDSS score progression [30,31].

Table 1 summarizes the significance of demographic factors for the prognosis of disease progression in the early stages of MS.

### 3.2. Clinical Factors

Clinical factors are among the most important aspects in predicting the course of multiple sclerosis, especially in the first years after onset. The primary progressive form is consistently recognized as one of the strongest adverse prognostic factors and is associated with a faster increase in disability progression compared to the relapsing-remitting form [10,18,34].

High relapse activity in the early stages of the disease is one of the best-documented predictors of further progression. Frequent relapses in the first year or first five years of the disease, especially if accompanied by incomplete remission, significantly increase the risk of rapidly reaching subsequent EDSS thresholds. Although there are cases when high EDSS during first relapse can be associated with better prognosis, combined with female sex, and younger age [21]. A particularly unfavorable prognostic factor is the persistence of clinical and radiological activity despite the use of disease-modifying treatment [10,35,36]. Early PIRA after a first demyelinating event is quite common and suggests an unfavorable long-term prognosis [37].

The nature of the initial symptoms is also prognostically significant. The onset of the disease in the form of isolated optic neuritis, a long interval between relapses, and complete remission after the first episode are usually associated with a better prognosis. In contrast, pyramidal, cerebellar, and brainstem symptoms, as well as sphincter dysfunction at the onset of the disease, are associated with a more aggressive course and faster progression of disability [18,19,28,38].

A polysymptomatic onset of the disease is considered one of the strongest adverse clinical factors. Simultaneous involvement of multiple functional systems reflects greater disease severity and is associated with faster achievement of disability milestones and a higher risk of transition to a secondary progressive form [18,36,39]. Early-stage prognostic stratification in multiple sclerosis continues to be an unresolved issue. Application of machine learning (ML) has proven highly effective for predictive analysis. Implementing ML algorithms showed EDSS at 24 months, age at symptom onset and disease duration at baseline as a short-term PIRA predictor in newly diagnosed pwMS [40].

Table 2 summarizes the significance of clinical factors for the prognosis of disease progression in MS.

### 3.3. Neuroimaging Factors

Magnetic resonance imaging plays a key role in assessing the prognosis in patients with multiple sclerosis. The number of hyperintense lesions in T2 sequences at the onset of the disease correlates with the risk of long-term disability progression. The strength of this association varies substantially across cohorts, with effect sizes generally modest and influenced by follow-up duration and treatment exposure [43]. A particularly poor prognosis is associated with the presence of numerous demyelinating lesions, especially when their number exceeds ten, although this threshold should be interpreted as probabilistic rather than deterministic [7,19,44].

Localization of lesion is a significantly important prognostic feature. Subtentorial, brainstem, and spinal lesions are associated with a higher risk of conversion to clinically definite MS and faster progression of disability [45]. Nevertheless, inter-study reproducibility is limited by heterogeneous MRI protocols and differing definitions of lesion involvement [46]. Contrast activity in MRI scans, especially if persistent in the first months of treatment, is a marker of treatment failure and increased risk of further disease activity [47].

Increasing attention is being paid to markers of chronic inflammatory activity, such as paramagnetic rim lesions (PRL) and slowly expanding lesions (SEL). Their presence is associated with a more aggressive course, faster onset of disability, and a higher risk of relapse-independent progression [48,49]. These markers are currently studied mainly in specialized centers, and their routine clinical applicability remains constrained by technical requirements and limited standardization [50,51].

Brain and spinal cord atrophy, including accelerated cervical spinal cord atrophy, is another important prognostic factor, strongly correlated with both motor disability and cognitive impairment [52]. Importantly, while atrophy measures show relatively larger effect sizes compared to focal lesion metrics, their use in individual prognostication is still limited by variability in acquisition and post-processing methods [53].

The significance of radiological prognostic factors is summarized in Table 3.

### 3.4. Laboratory Factors

The best-established laboratory prognostic factor remains the presence of oligoclonal bands (OCBs) in cerebrospinal fluid, indicating intrathecal immunoglobulin synthesis. Their presence is associated with a higher risk of conversion from clinically isolated syndrome to clinically definite MS and with faster disease progression [55]. However, their value for predicting long-term disability progression is less pronounced and should not be considered in isolation [56,57].

In recent years, the importance of biomarkers of neurodegeneration and inflammatory activity has been growing. The concentration of light neurofilaments in serum and cerebrospinal fluid correlates with disease activity, the number of MRI lesions, and the risk of future progression. Most evidence derives from observational studies, and proposed cut-off values vary considerably between assays and populations. Free kappa light chains are a promising alternative or supplement to oligoclonal bands, and their elevated index is associated with a higher risk of PIRA, but their added prognostic value beyond established markers remains under investigation [58,59].

Similarly, GFAP is emerging as a marker of astrocytic pathology and progression, although current data are still insufficient to support its routine use in early risk stratification [58,59].

Despite promising results, most laboratory biomarkers have not yet been standardized to the extent that they can be routinely used in clinical practice, highlighting the need for further validation studies.

Table 4 provides an overview of the significance of laboratory prognostic factors.

### 3.5. Clinical Significance

The assessment of prognostic factors allows the identification of patients at high risk of aggressive MS at an early stage of the disease and may provide a basis for individualizing the therapeutic strategy, including consideration of early use of highly effective therapies. Table 2, Table 3 and Table 4 summarize key clinical, MRI, and laboratory factors associated with unfavorable prognosis and illustrates their potential integration into a practical risk-stratification approach. The proposed framework is intended as an interpretative aid rather than a validated clinical algorithm.

## 4. Disease-Modifying Therapy

The decision to choose a disease-modifying therapy (DMT) in patients with newly diagnosed multiple sclerosis is one of the key elements of clinical management and has a significant impact on long-term prognosis. Contemporary therapeutic strategies are based mainly on two approaches: the escalation model, which assumes a gradual increase in the effectiveness of treatment in the event of persistent disease activity, and the strategy of early use of high-efficacy therapy (HET) [4,5,65].

In recent years, there has been growing interest in the strategy of early use of highly effective therapy, especially in patients with features of aggressive disease progression. Available data from observational studies and real-world analyses suggest potential benefits in reducing inflammatory activity and slowing disability progression. It should be emphasized, however, that these findings are not derived from randomized comparative trials, and causal inferences should therefore be made with caution [66,67,68,69].

The key challenge remains the accurate identification of patients who will benefit most from early HET. As demonstrated in previous sections, factors supporting a more intensive therapeutic strategy include high relapse activity in the early years of the disease, incomplete remission after relapses, polysymptomatic onset, unfavorable MRI features (multiple T2 lesions, spinal cord lesions, PRLs, SELs), and elevated levels of neurodegenerative biomarkers. The use of these parameters in clinical practice allows for more informed and individualized therapeutic decisions [4,6,7].

Despite the growing evidence supporting early use of HET, there is still a lack of randomized clinical trials clearly comparing the two strategies in defined risk groups. In addition, highly effective therapies are associated with a potentially higher risk of adverse events, which requires careful assessment of the benefit-risk ratio and consideration of patient preferences.

The choice of therapeutic strategy in multiple sclerosis should be based on an integrated assessment of prognostic factors, the patient’s clinical characteristics, and the potential benefits and risks associated with available therapies. Early personalization of treatment, based on reliable risk stratification, is currently one of the most important directions for optimizing care for patients with MS [4,7,70,71].

## 5. Discussion

Multiple sclerosis remains a disease with a distinctly heterogeneous clinical course, which significantly hinders prognosis and treatment optimization in the early stages of the disease. This review confirms that the prognosis for patients with newly diagnosed MS is determined by a complex interaction of demographic, clinical, neuroimaging, and laboratory factors, none of which has sufficient predictive power alone. Only their combined analysis allows for more reliable risk stratification and more informed therapeutic decisions.

Data on the natural course of the disease indicate that, despite the traditional division of MS into relapsing and progressive forms, the disease is a continuum of inflammatory and neurodegenerative processes [2]. Early inflammatory activity, manifested by frequent relapses and high activity on MRI scans, is associated with faster achievement of disability milestones [11]. At the same time, numerous long-term studies suggest that the rate of disability progression is largely dependent on the patient’s age, which supports the concept of common mechanisms underlying disease progression, regardless of the clinical phenotype at the time of diagnosis [8].

Among clinical factors, the course of the disease in the first few years is of particular prognostic significance. High relapse activity, incomplete remission after relapses, and a polysymptomatic onset consistently correlate with a poorer prognosis [10,18,35,36,39]. These observations have direct practical implications, as they emphasize the need for close monitoring of patients in the early stages of the disease and rapid assessment of the effectiveness of the treatment used. The nature of the initial symptoms also remains important, with involvement of the pyramidal, cerebellar, or brainstem systems signalling a higher risk of aggressive disease progression [18,28,38,45].

Magnetic resonance imaging remains one of the key prognostic tools in MS. The number and location of T2 lesions, the presence of contrast enhancement, spinal cord lesions, and features of chronic inflammatory activity, such as paramagnetic rim lesions (PRLs) or slowly expanding lesions (SELs), are associated with a higher risk of disability progression and the development of progression independent of relapse activity (PIRA) [19,44,45,47,48,49,52]. Of particular importance is the growing significance of markers of chronic inflammatory activity, which allow for better capture of the neurodegenerative component of the disease, which is less well reflected by classic MRI parameters.

Laboratory biomarkers are playing an increasingly important role as a supplement to clinical and neuroimaging assessments. The presence of oligoclonal bands in cerebrospinal fluid remains one of the strongest predictors of conversion from clinically isolated to clinically definite MS. In turn, light neurofilaments, free kappa light chains, and GFAP are promising markers of disease activity and neurodegenerative progression. The combined use of several biomarkers seems particularly interesting, as it may increase the accuracy of predicting the risk of PIRA and long-term disability [55,58,61,64]. However, it should be emphasized that the lack of standardization of measurement methods and clear cut-off points still limits their routine clinical use. Early-stage prognostic stratification in multiple sclerosis continues to be an unresolved issue. Application of machine learning (ML) has proven highly effective for predictive analysis. Implementing ML algorithms showed EDSS at 24 months, age at symptom onset and disease duration at baseline as a short-term PIRA predictor in newly diagnosed pwMS [40].

Summary of prognostic factors with level of evidence, potential biases and clinical interpretation is presented in Table 5. The proposed framework is intended as an interpretative aid rather than a validated clinical algorithm.

From a therapeutic perspective, the collected data supports the concept of early identification of patients with an unfavorable prognostic profile as a key element in optimizing treatment. A growing number of observational studies and real-world evidence analyses suggest that early use of highly effective therapies may be more effective in reducing inflammatory activity and slowing the progression of disability compared to an escalation approach. At the same time, the lack of randomized trials directly comparing these strategies in clearly defined risk groups highlights the need for cautious interpretation of the available data. The absence of validated prognostic algorithms and randomized trials, comparing treatment strategies based on early risk profiles represents a major gap in the literature. While observational data consistently suggest benefits of early high-efficacy therapy, these results should be interpreted as hypothesis-generating rather than definitive, underscoring the need for prospective, controlled studies.

A limitation of this review is the heterogeneity of the studies analyzed, which includes different patient populations, different definitions of aggressive disease course, and different endpoints. Most prognostic data originate from observational cohorts and real-world registries, while randomized controlled trials addressing prognostic stratification are largely lacking. Registry-based studies, although valuable for long-term outcomes, may be affected by treatment-selection bias and incomplete adjustment for confounding variables. Nevertheless, the consistency of observations regarding the importance of early disease activity and neuroimaging features reinforces their clinical value.

Current evidence indicates that effective prediction of the course of multiple sclerosis requires a multidimensional approach integrating clinical, imaging, and laboratory data. Such an approach provides the basis for more precise personalization of treatment and may contribute to real modification of the natural course of the disease.

### Prognostic Models and Predictive Tools

Several composite prognostic models and scoring systems have been proposed to predict disease progression in multiple sclerosis, incorporating clinical, MRI, and, more recently, biomarker data. In addition, machine-learning and artificial intelligence–based approaches have shown promising results in retrospective datasets [72,73,74,75].

However, most existing models lack external validation, are based on selected cohorts, or require data not routinely available in clinical practice. As a result, no prognostic tool is currently recommended for widespread use in individual patient management. This limits the immediate clinical applicability of algorithm-based prediction and supports the continued use of integrated clinical judgment informed by multiple prognostic domains [76].

## 6. Conclusions and Directions for Future Research

The course of multiple sclerosis is characterized by pronounced interindividual heterogeneity, driven by the interaction of inflammatory and neurodegenerative mechanisms. This review provides an updated and integrated overview of early prognostic factors, emphasizing their relevance for clinical decision-making at the earliest stages of the disease. High relapse activity in the first years, incomplete recovery after relapses, polysymptomatic onset, primary progressive disease course, and unfavorable MRI features represent the most consistently confirmed adverse prognostic factors. Emerging imaging and laboratory markers further refine risk assessment but should be interpreted within the context of their current methodological limitations.

An integrated assessment of prognostic factors allows the identification of patients at high risk of aggressive disease progression and may form the basis for individualizing the therapeutic strategy, including consideration of early use of highly effective therapies. Current risk stratification remains probabilistic rather than deterministic, reflecting the limitations of available prognostic evidence. Laboratory biomarkers such as neurofilament light chain, free kappa light chains, and GFAP offer promising insights into disease activity and progression. However, their main value at present lies in complementing established clinical and MRI markers rather than replacing them.

From a therapeutic perspective, early identification of patients with an unfavorable prognostic profile may inform treatment intensity. Importantly, current evidence supporting early high-efficacy therapy is derived primarily from observational and real-world studies, highlighting the need for cautious interpretation. The main innovative contribution of this review lies in its integrative and translational approach, synthesizing diverse prognostic domains into a clinically meaningful framework rather than presenting isolated predictors. Future research should prioritize the validation of multidimensional prognostic models and the design of prospective trials evaluating treatment strategies guided by early risk stratification.

## Figures and Tables

**Table 1:** Demographic prognostic factors.

Prognostic Factor	Study Design	Study Size	Enrolment Period	Results	References
**Gender**	Retrospective, multi-center, cohort study	11,570 pwRRMS881 pwPPMS	1951–2012	Relapse frequency 17.7% higher in females than males	[20]
	Retrospective-prostective, cohort study	1562 pwRRMS282 pwPPMS	1957–2006	Females have more benign course and take longer time to reach higher disability score	[8]
	Retrospective, multi-center, cohort study	1099 untreated pwMS	1972–1984	Males have slightly higher risk of developing SPMS than females	[16]
	Retrospective- prospective, cohort study	2484 pwMS	1988–2008	Males reached SPMS more rapidly from onset and at a younger age	[17]
	Prospective, cohort study	224 pwMS	1983–1996	No prognostic significance of gender	[18]
	Prospective, cohort study	1058 pwMS	1995–2013	Occurrence of a CIS is higher in females, gender seems not to be a risk factor for further relapse or the development of disability	[19]
**Age at onset**	Retrospective- prospective, cohort study	1562 pwRRMS282 pwPPMS	1957–2006	The younger the onset, the younger the age at assignment of disability milestones	[23]
	Prospective, single-centre cohort study	661 pwMS	2000–2020	The younger age at onset the higher risk of reaching SPMS earlier and having higher EDSS	[24]
	Retrospective cohort study	19,318 pwMS	1993–2021	Age at onset > 40 years is associated with higher risk of disability	[22]
**Race**	Retrospective, cohort study, real-word data	5602 pwMS	1996–2003	African American patients were more likely to have greater disability with increased disease duration	[27]
	Retrospective, cohort study	802 pwMS	1998–2002	African American patients with MS have a more aggressive disease course compared to Caucasian Americans	[26]
	Retrospective, cohort study	303 pwMS	1980–2014	Benign course was significantly more frequent among Caucasians when compared to Afrodescendants	[28]
**Comorbidities**	Retrospective-prospective, cohort study, real-word data	8983 pwMS	1986–2006	One or more vascular comorbidities at diagnosis had an increased risk of disability, and risk increased with the number of vascular conditions reported	[29]
	Population-based, case–control study	1571 pwMS, 3371 controls	2005–2010	Subjects whose BMI exceeded 27 kg/m^2^ at age 20 had a two-fold increased risk of developing MS compared with normal weight subjects	[30]
	Meta-analysis, 11 studies	1945 smokers and 2085 non-smokers pwMS	2017	Ever smoking was significantly associated with increased EDSS and disability progression	[31]
	Retrospective, cohort study	2083 pwMS	2008–2012	Older age, higher BMI, lower median income, ever smokers were associated with higher disability.	[32]
	Meta-analysis -241 studies		2024	Higher age, longer disease duration, smoking and vascular comorbidities were associated with poor long-term prognosis	[33]

**Table 2:** Clinical prognostic factors.

Prognostic Factor	Study Design	Study Size	Enrolment Period	Results	References
**Relapses**	Retrospective, multicenter, cohort study	19,318 pwMS	2016–2021	Risk factors for SPMS were a multifocal onset, an age at onset > 40 years, higher baseline EDSS score and a higher number of relapses	[22]
	Prospective, cohort study	784 pwMS	2013–2019	A high number of pretreatment relapses was only associated with an increased risk of disease activity in females	[41]
	Retrospective, cohort study	134 pwMS	2016–2020	Female sex, younger age, and higher EDSS at first relapse are associated with higher chance of EDSS improvement	[21]
	Prospective, multi-center, cohort study	1280 pwMS	1990–2014	Clinical relapses, during the first year of treatment with interferon-β indicates significant risk of treatment failure and EDSS worsening in the short term	[35]
**Functional systems involvement**	Retrospective- prospective, cohort study	308 pwMS	1950–1964	Early predictors were unable to predict the rate of progression. Late predictors: a shorter time from onset to secondary progression and a higher number of functional systems involved at onset of progression predicted a faster progression rate.	[36]
	Retrospective, multi-center, cohort study	1099 untreated pwMS	1972–1984	Persisting deficits in brainstem, cerebellar or cerebral systems, a higher frequency of attacks in the first 2 years after onset of disease, a short first interattack interval are associated in faster disability progression	[38]
	Retrospective, cohort study	2934 pwMS	1981 (prevalence day)	Older age of onset, progressive disease course, onset symptoms that were multiple, pyramidal or cerebellar, and a short interval between onset and first relapse were associated with higher EDSS	[39]
	Retrospective, multi-center, cohort study, ML analysis	719 pwMS	2000–2021	EDSS at 24 months, age at symptom onset and disease duration at baseline were features with the highest predictive impact of PIRA	[40]
	Retrospective-prospective, cohort study, RWD	485 pwMS	2016–2026	Multifocal onset, delayed treatment initiation, presence of spinal cord lesions and oligoclonal bands were identified as risk factors of not achieving NEDA-3 status.	[42]
	Mea analysis-241 studies		2024	Motor, cerebellar, sphincter symptoms and cognitive dysfunction were strongly associated with a poor long-term prognosis	[33]
**Progressive onset**	Retrospective, multi-center, cohort study	1099 untreated pwMS	1972–1984	Patients with primary progressive MS were associated with higher disability	[38]
	Prospective, cohort study	224 untreated pwMS	1983–1990	In progressive MS a greater number of functional systems involved at onset as well as higher residual deficits in pyramidal, visual, sphincteric and cerebellar systems were other factors predictive of a poor outcome	[18]
PIRA	Prospective, cohort study	1128 pwMS	1994–2021	Early PIRA is associated with higher EDSS increase rate yearly	[37]

**Table 3:** Radiological prognostic factors.

Prognostic Factor	Study Design	Study Size	Enrolment Period	Results	References
**T2 and Gd(+) lesions**	Prospective, cohort study	1015 pwMS	1995–2013	10 or more T2 brain lesions on magnetic resonance increased the risk of MS and disability	[19]
	Prospective, cohort study	1015 pwMS	1995–2013	10 or more T2 brain lesions on magnetic resonance increased the risk of MS and disability	[19]
	Retrospective, cohort, double-center study	687 pwMS	2009–2021	>9 T2 lesions and Gd(+) lesions on baseline MRI scan were predictive of RAW. Presence of ≥1 spinal cord lesion on baseline MRI scan Was predictive of PIRA	[7]
	Meta-analysis of randomized trials (31 studies)	18,901 pwMS	2008–2012	Analysis of trials showed that the effects on MRI lesions over short follow-up periods (6–9 months) can also predict the effects on relapses over longer follow-up periods (12–24 months)	[47]
**SELs**	Prospective, cohort study	299 pwMS	2013–2017	Larger number of SELs correlated with progressive form of MS	[49]
	Prospective, cohort study	52 pwMS	2012–2021	Higher proportion of SELs in baseline MRI is a predictor of EDSS worsening	[54]
**Lesion volume and localization of lesions**	Prospective, cohort study	140 pwCIS	1984–2008	Lesion volume correlated with disability after 20 years. Rate of lesion growth is 3× higher in those who develop SPMS	[44]
	Prospective, cohort study	246 pwCIS	1995–2008	Infratentorial lesions (cerebellar and brainstem) were associated with the risk of conversion to cdMS and disability progression	[45]
**PRLs**	Prospective, cohort study	209 pwMS	2012–2018	Individuals with 4 rim lesions or more reached motor and cognitive disability at an earlier age.	[48]
	Retrospective, cohort study	133 pwMS	2011–2013	PRLs are associated with more frequent future clinical relapses and greater long-term, relapse-independent disability progression.	[50]
	Prospective, observational, cross-sectional	31 pwMS	2019–2021	PRL presence at baseline was associated with confirmed EDSS progression	[51]
**Brain and spinal cord atrophy**	Prospective, observational study	39 pwMS	2024	Atrophy of deep gray matter (thalamus, putamen) and cerebellar structures is associated with cognitive impairment	[53]
	Meta-analysis	20,621 pwMS	2023	For cognitive impairment in MS patients, reliable predictors include cortical gray matter volume, brain atrophy, lesion characteristics, white matter lesion volume, thalamic volume, and corpus callosum density.	[43]
	Prospective, cohort study	50 pwPPMS	1999–2001	Early brain atrophy and spinal cord involvement are associated with progression in long-term outcome	[52]

**Table 4:** Laboratory prognostic factors.

Prognostic Factor	Study Design	Study Size	Enrolment Period	Results	References
**Oligoclonal bands**	Metanalysis	12,253 pwMS and 2685 pwCIS	2012	OCBs positivity strongly predicts conversion from CIS to MS.	[55]
	Retrospective, registry-based study	7322 pwMS	2018	OCBs positivity is associated with higher risk of unfavorable outcomes: both reaching disability milestones and SPMS conversion.	[60]
	Prospective, cohort study	44 pwMS	2011–2015	There are no remarkable cognitive differences between lipid-specific—OCMB− and lipid-specific—OCMB+ patients in the early stages of MS.	[56]
	Prospective, cohort study	193 pwMS	1994–2020	Intrathecal immunoglobulin (Ig) M synthesis predicts an aggressive MS at disease onset, especially when detected as lipid-specific OCMBs	[57]
**Kappa free light chain (KFLC)**	Retrospective, cohort study	131 pwCIS and pwMS	2013–2020	high KFLC index at baseline is predictive of PIRA, evidence of disease activity, and overall worse prognosis in MS.	[61]
	Cross-sectional observational study	78 pwMS	2015–2022	CSF FKLC and kappa-Index were predictors of verbal memory impairment.	[62]
**sGFAP and sNFL**	Prospective, cohort, longitudinal study	355 pwMS	2012–2022	sGFAP is a prognostic biomarker for future PIRA	[58]
	Retrospective, cohort study	133 pwMS	2014–2017	Serum NfL and GFAP do not seem to accurately predict MS outcome in the long term.	[59]
	Prospective, cohort, longitudinal study	257 pwMS	2007–2021	Higher sGFAP levels, but not sNfL levels, were associated with higher risk of 6mCDP	[63]
Serum NfL level	Case–control study	1313 pwMS, 5390 controls	1992–2020 (control group) 2012–2021 (pwMS group)	Patients with higher sNfL Z-score were at risk of EDSS worsening	[64]

**Table 5:** Factors associated with unfavorable prognosis—risk-stratification approach.

Prognostic Domain	Factor	Strength of Evidence	Main Limitations	Clinical Interpretation
**Demographic**	Older age at onset	Moderate–strong (cohort studies)	Age-related confounding with progression mechanisms	Associated with faster attainment of disability milestones
	Male sex	Weak–moderate (heterogeneous data)	Inconsistent results across cohorts	May indicate higher risk of progression in selected populations
**Clinical**	High relapse activity in first 1–5 years	Strong (long-term observational cohorts)	Relapse definitions vary; treatment effects	One of the most robust early predictors of aggressive course
	Incomplete recovery after relapses	Strong	Subjective assessment; EDSS limitations	Reflects early irreversible tissue damage
	Polysymptomatic onset	Moderate–strong	Retrospective bias	Marker of higher initial disease burden
	Primary progressive onset	Strong	Limited therapeutic comparators	Consistently associated with rapid disability progression
**MRI**	High T2 lesion load (>10 lesions)	Moderate	Thresholds not absolute; modest effect sizes	Probabilistic indicator of higher long-term risk
	Spinal cord lesions	Strong	MRI protocol variability	Associated with faster disability accumulation
	Gadolinium-enhancing lesions	Moderate	Reflects short-term activity	Marker of ongoing inflammatory activity
	Paramagnetic rim lesions (PRL)	Emerging	Limited availability; lack of standardization	Marker of chronic active inflammation
	Slowly expanding lesions (SEL)	Emerging	Research-based definitions	Associated with relapse-independent progression
	Brain/spinal cord atrophy	Moderate–strong	Post-processing variability	Reflects neurodegeneration rather than inflammation
**Laboratory**	CSF oligoclonal bands	Strong(meta-analyses)	Limited long-term disability prediction	High risk of conversion from CIS to MS
	Neurofilament light chain (serum/CSF)	Moderate–strong	Assay variability; cut-off uncertainty	Marker of axonal damage and future activity
	Free kappa light chains	Emerging–moderate	Limited external validation	Potential predictor of PIRA
	GFAP	Emerging	Early-stage evidence	Marker of astrocytic pathology and progression

## Data Availability

Not applicable.
